# Heparan Sulfate Proteoglycans Mediate Interstitial Flow Mechanotransduction Regulating MMP-13 Expression and Cell Motility via FAK-ERK in 3D Collagen

**DOI:** 10.1371/journal.pone.0015956

**Published:** 2011-01-05

**Authors:** Zhong-Dong Shi, Hui Wang, John M. Tarbell

**Affiliations:** Department of Biomedical Engineering, The City College of New York, The City University of New York (CUNY), New York, New York, United States of America; University of Pittsburgh, United States of America

## Abstract

**Background:**

Interstitial flow directly affects cells that reside in tissues and regulates tissue physiology and pathology by modulating important cellular processes including proliferation, differentiation, and migration. However, the structures that cells utilize to sense interstitial flow in a 3-dimensional (3D) environment have not yet been elucidated. Previously, we have shown that interstitial flow upregulates matrix metalloproteinase (MMP) expression in rat vascular smooth muscle cells (SMCs) and fibroblasts/myofibroblasts via activation of an ERK1/2-c-Jun pathway, which in turn promotes cell migration in collagen. Herein, we focused on uncovering the flow-induced mechanotransduction mechanism in 3D.

**Methodology/Principal Findings:**

Cleavage of rat vascular SMC surface glycocalyx heparan sulfate (HS) chains from proteoglycan (PG) core proteins by heparinase or disruption of HS biosynthesis by silencing *N*-deacetylase/*N*-sulfotransferase 1 (NDST1) suppressed interstitial flow-induced ERK1/2 activation, interstitial collagenase (MMP-13) expression, and SMC motility in 3D collagen. Inhibition or knockdown of focal adhesion kinase (FAK) also attenuated or blocked flow-induced ERK1/2 activation, MMP-13 expression, and cell motility. Interstitial flow induced FAK phosphorylation at Tyr925, and this activation was blocked when heparan sulfate proteoglycans (HSPGs) were disrupted. These data suggest that HSPGs mediate interstitial flow-induced mechanotransduction through FAK-ERK. In addition, we show that integrins are crucial for mechanotransduction through HSPGs as they mediate cell spreading and maintain cytoskeletal rigidity.

**Conclusions/Significance:**

We propose a conceptual mechanotransduction model wherein cell surface glycocalyx HSPGs, in the presence of integrin-mediated cell-matrix adhesions and cytoskeleton organization, sense interstitial flow and activate the FAK-ERK signaling axis, leading to upregulation of MMP expression and cell motility in 3D. This is the first study to describe a flow-induced mechanotransduction mechanism via HSPG-mediated FAK activation in 3D. This study will be of interest in understanding the flow-related mechanobiology in vascular lesion formation, tissue morphogenesis, cancer cell metastasis, and stem cell differentiation in 3D, and also has implications in tissue engineering.

## Introduction

In living tissues, many cell types including smooth muscle cells (SMCs), fibroblasts, bone cells, and tumor cells are exposed to interstitial fluid flow. Interstitial flow can modulate many cellular processes in a 3-dimensional (3D) microenvironment including proliferation, apoptosis, differentiation, and migration [1]–[5]. Interstitial flow therefore plays important roles in tissue physiology and pathology. For example, during the early stages of vascular injury, elevated interstitial flow has been hypothesized to contribute to neointima formation by affecting vascular wall cell phenotype and motility [1], [2], [6]–[8].

To investigate effects of interstitial flow on biology of tissue interstitial cells including vascular wall cells, bone cells, and tumor cells, application of fluid flow shear stress to cells cultured in 2D has been widely used [6], [9]–[11]. It is now well recognized that culturing cells in a 3D extracellular matrix (ECM) cell culture better mimics in vivo cell physiology than traditional 2D planar culture [12]. It has been reported that interstitial flow can induce cytokine release, cell migration, capillary morphogenesis, and stem cell differentiation in 3D environments [1], [3], [7], [13]–[15]. However, the mechanism by which cells in 3D sense interstitial flow and convert this stimulation into cellular responses (mechanotransduction) has not yet been elucidated. Shear stress-induced mechanotransduction in endothelial cells (ECs) in 2D has been well studied [16], [17]. Cells embedded in a 3D ECM have different patterns of cell-matrix adhesions [12] and elongated morphologies compared to 2D [18], which might give rise to different mechanotransduction mechanisms. Therefore, it is necessary to determine the mechanosensors for cells in 3D when exposed to interstitial flow.

In 2D studies, it has been suggested that cell surface glycocalyx components are responsible for sensing fluid shear stress on vascular ECs [19]–[21] and SMCs [9]. The surfaces of eukaryotic cells are decorated with a layer of glycocalyx. The glycocalyx consists primarily of proteoglycan (PG) core proteins that are incorporated into the cell membrane and several covalently bound glycosaminoglycan (GAG) chains that extend into extracellular space [9]. Heparan sulfate (HS), chondroitin sulfate, and hyaluronan are the dominant GAGs on most cell surfaces. Glycocalyx components, especially heparan sulfate proteoglycans (HSPGs), have been shown to play important roles in cellular recognition and signaling, cell growth, adhesion, spreading, and migration, regulating development, tumorigenesis, and vasculogenesis [22]–[25]. Although, in 2D, the role of cell surface glycocalyx component HSPGs in flow-induced mechanotranduction has been extensively studied [9], [19]–[21], and also we have shown recently that HSPGs play a role in fluid flow modulation of SMC marker expression in both 2D and 3D [2], the role of HSPGs in flow sensing in 3D has not been well elucidated.

Focal adhesion kinase (FAK) is a widely expressed cytoplasmic protein tyrosine kinase located in integrin-mediated focal adhesions that regulates integrin signaling. FAK is a major mechanosensitive kinase that can be rapidly activated by a variety of mechanical stimuli and plays an important role in control of cell adhesion and migration [26], [27]. It has been suggested that HSPGs (such as syndecan-1 and -4) can act cooperatively with integrins in creating signals for cell spreading and for assembly of focal adhesion plaques and stress fibers [28]–[31]. HSPGs themselves can also tether to ECM binding domains with HS chains serving as secondary cell-matrix adhesions [22]. When cells are plated on fibronectin, syndecan-4 can associate with FAK through paxillin and thus has the potential to mediate signaling events parallel to integrins [32]. In 2D, it is well known that HSPGs on the apical surface of cells can act as mechanosensors mediating the transduction of fluid shear stress into biochemical responses [20], [33], [34]. On the basal side, similar to integrins, syndecan HSPGs can bind to the substrate and interact with the cytoskeleton to modulate FAK and ERK activation [35], [36], suggesting that HSPG-mediated attachments are capable of providing separate mechanosignaling pathways.

We have shown previously that interstitial flow can activate an ERK1/2-c-Jun signaling cascade leading to increased expression of rat MMP-13 (rat interstitial collagenase should be designated as MMP-13, not as MMP-1 in our previous articles [1], [7]), which in turn promotes rat vascular SMC, fibroblast and myofibroblast migration in 3D collagen [1], [7]. Based on this background, we now demonstrate, for the first time, that, with contributions from integrins, cell surface HSPGs are mechanosensors for sensing interstitial flow that leads to activation of the FAK and ERK signaling cascade and upregulation of MMP expression and cell motility in 3D.

## Results

### Interstitial flow-induced MMP-13 expression and SMC motility in 3D collagen depend on HSPGs

**Figure 1:**
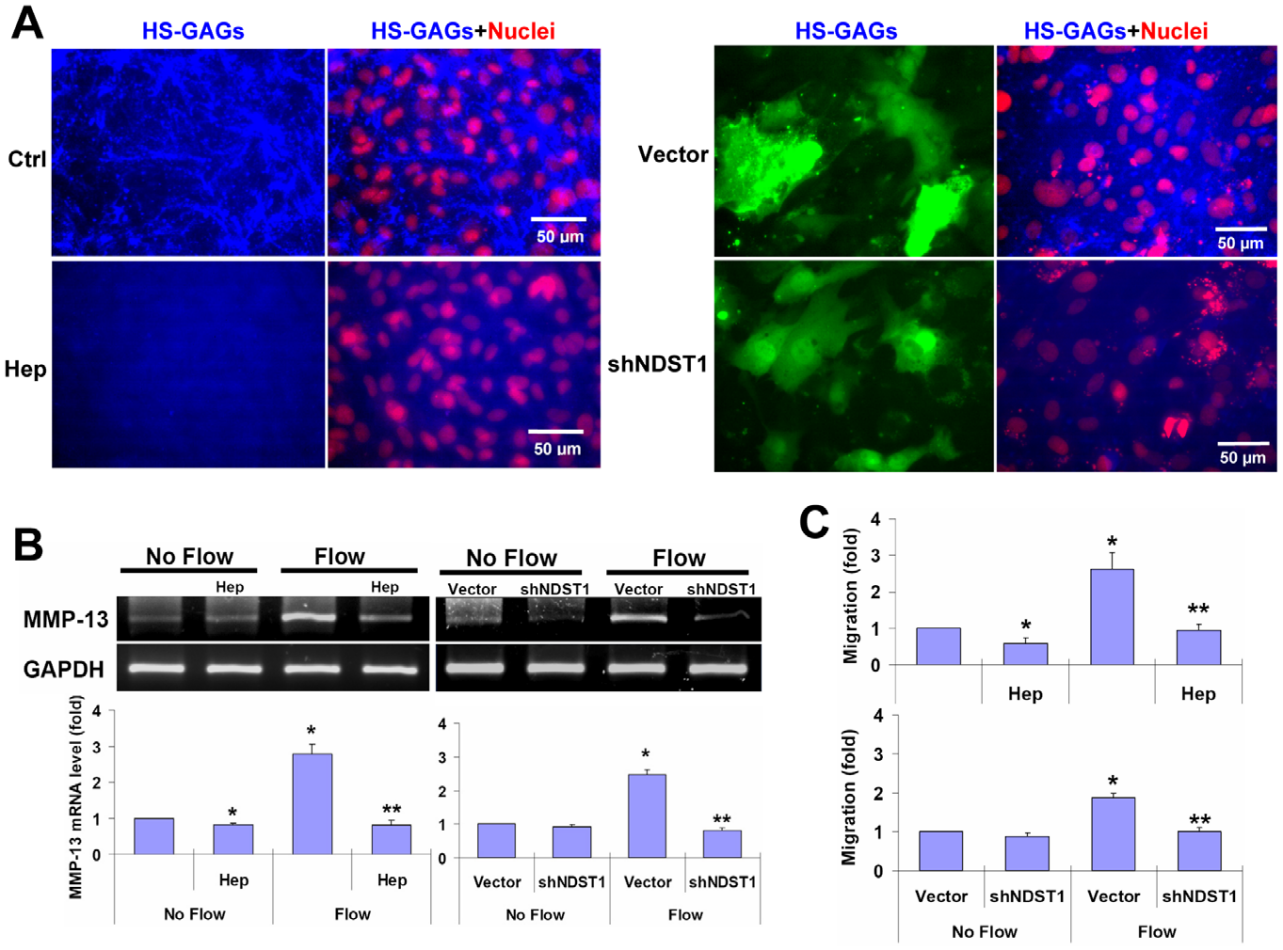
Interstitial flow promotes MMP-13 expression and cell motility dependent on HSPGs. (**A**) Immunostaining to show that HSPGs are present on the surfaces of cells cultured in 2D, and both Hep and shNDST1 successfully eliminated HS-GAGs (See Figure S1 for more information). Blue: HS-GAGs; Red: nuclei; Green: GFP. (**B**) Disruption of HS-GAGs abolished interstitial flow-induced MMP-13 expression (gel panel data: RT-PCR; bar graph: RT-qPCR). (**C**) Interstitial flow-induced cell motility was abolished by disruption of HSPGs. For Hep treatments, after 24 h of cell spreading in collagen, growth media was replaced with either fresh media or 6.7 IU/L Hep, and then incubated for 2 h, followed by 4.5 h of exposure to interstitial flow. After exposed to flow, some cells in gels were subjected to RNA measurement or motility test. Bar graph data are presented as mean ± SEM, n = 3–4. * P<0.05 vs No-flow controls; ** P<0.05 vs Flow cases. HSPGs: heparan sulfate proteoglycans; GAG: glycosaminoglycan; Hep: heparinase III; shNDST1: NDST1 shRNA; GFP: green fluorescent protein.

HS-GAGs are abundantly presented on the surfaces of rat vascular SMCs, and can be substantially cleaved by a selective enzyme, heparinase III (Figure 1A left and Figure S1). This is consistent with our previous observations [2]. HS chain production can be effectively suppressed by silencing *N*-deacetylase/*N*-sulfotransferase 1 (NDST1), an enzyme that modulates HS biosynthesis with short hairpin RNA (shNDST1) (Figure 1A right and Figure S1). To investigate whether the HSPGs were responsible for sensing 3D interstitial flow, heparinase and shNDST1 were used to disrupt cell surface HSPGs. Cleavage of HS-GAGs by heparinase completely abolished flow-induced MMP-13 expression (Figure 1B), resulting in a significant reduction in flow-induced cell motility (Figure 1C). Heparinase also reduced MMP-13 expression and cell motility in the no-flow control case. Knockdown of NDST1 by shNDST1 abolished the augmentation of MMP-13 expression and cell motility induced by interstitial flow (Figures 1B and 1C). It appears that shNDST1 and heparinase had similar effects on MMP-13 expression and cell motility. Previously we have shown that inhibition of MMP-13 or ERK1/2 does not attenuate the baseline migration (no-flow control cases) in SMCs [1], [7]. Therefore, in this study, inhibition of cell motility after cleavage of HSPGs by heparinase was not due to reduced MMP-13. It probably was due to the reduced cell-matrix adhesion assembly and disassembly after removal of HSPGs, since HSPGs can enhance formation of cell-matrix adhesions and stress fibers [28]–[31], [35].

### Interstitial flow-induced MMP-13 expression and cell motility in 3D depend on FAK

**Figure 2:**
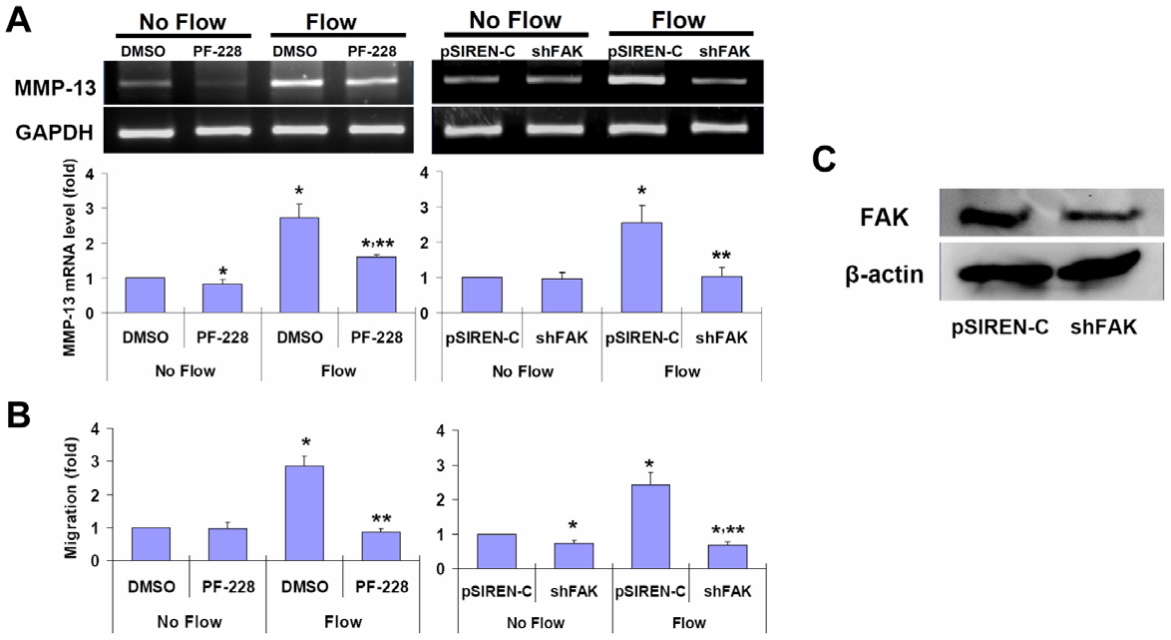
Interstitial flow promotes MMP-13 expression and cell motility dependent on FAK. (**A**) A FAK inhibitor (PF-228) or FAK shRNA (shFAK) abolished flow-induced MMP-13 expression (gel panel data: RT-PCR; bar graph data: RT-qPCR). (**B**) Flow-induced cell motility was abolished by inhibition of FAK. (**C**) Western blots show FAK protein expression was dramatically inhibited by shFAK. Cells were incubated with DMSO or 10 µM PF-228 in medium for 2 h and then exposed to flow for 4.5 h. Bar graph data are presented as mean ± SEM, n = 3–4. * P<0.05 vs No-flow controls; ** P<0.05 vs Flow cases. FAK: focal adhesion kinase; shFAK: FAK shRNA; pSIREN-C: control for shFAK.

To investigate whether FAK was involved in flow-induced MMP-13 expression and cell motility in 3D, a FAK inhibitor (PF-228) and FAK shRNA (shFAK) were used to inhibit FAK. The efficacy of FAK knockdown was evaluated by Western blotting (Figure 2C). PF-228 significantly attenuated but did not completely abolish flow-induced MMP-13 expression (Figure 2A) and completely abolished flow-enhanced cell motility (Figure 2B). With knockdown of FAK, flow-induced MMP-13 expression was completely inhibited (Figure 2A) and cell motility was completely abolished to a level even lower than the control case (Figure 2B). In the no-flow control cases, PF-228 slightly reduced MMP-13 expression but not cell motility, while shFAK significantly suppressed cell motility but not MMP-13 expression (Figures 2A and 2B). FAK shRNA reduces the total amount of FAK, possibly resulting in less FAK available for focal adhesion turnover and therefore less cell motility [37]. PF-228 selectively inhibits FAK phosphorylation at Tyr397, but does not affect the total amount of FAK. PF-228 inhibits cell migration concomitant with the inhibition of focal adhesion turnover [38]. Therefore, our data indicate that phosphorylation of FAK at Tyr397 is critical for flow-induced cell motility through adhesion turnover. Our data also suggest that FAK phosphorylation at other tyrosine residues may play an important role in MMP-13 expression, because FAK shRNA completely abolished flow-induced MMP-13 expression but PF-228 did not. This data again shows that downregulation of MMP-13 expression by PF-228 in the no-flow case did not attenuate baseline migration, which is consistent with our previous observations [1], [7].

### FAK and HSPGs mediate interstitial flow-induced ERK activation

**Figure 3:**
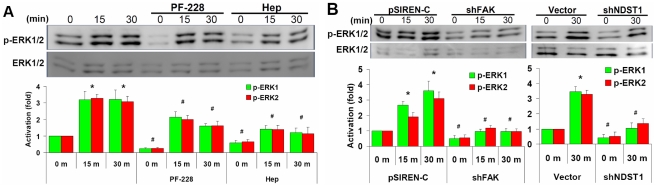
Interstitial flow-induced ERK activation depends on both FAK and HSPGs. (**A**) Western blots showed that PF-228 and Hep reduced ERK phosphorylation; Cells in gels were pretreated with 10 µM of PF-228 or 6.7 IU/L of Hep for 2 h followed by exposure to flow for 0, 15, or 30 min. (**B**) Western blots showed that shFAK or shNDST1 significantly reduced flow-induced ERK activation. Flow was induced for 0, 15, or 30 min. The gel panels are representative images from three independent experiments. Fold change values are the ratios of p-ERK1 and p-ERK2 over the total ERK1 and total ERK2, and then normalized to no-flow control case, shown in the bar graph. * P<0.05 vs time “0” in the non-treated control cases; # P<0.05 vs corresponding time points in the non-treated control cases.

We have previously demonstrated that interstitial flow-induced MMP-13 expression depends on ERK activation [7]. Above we showed that flow-induced MMP-13 upregulation also depends on both FAK and HSPGs. Therefore, we further investigated whether FAK and HSPGs regulate flow-induced ERK activation. Flow significantly stimulated ERK phosphorylation, and PF-228 dramatically reduced ERK activation in the no-flow control, and partially, but significantly, attenuated flow-induced ERK activation (Figure 3A), which is consistent with MMP-13 expression (Figure 2A). Knockdown of FAK substantially inhibited ERK activation in both no-flow and flow cases (Figure 3B). These results suggest that there must be FAK tyrosine sites other than Tyr397 that play a more dominant role in flow-induced ERK activation and downstream MMP-13 expression (Figure 2A). Cleavage of HSPGs by heparinase significantly inhibited ERK activation in both no-flow control and flow cases (Figure 3A), consistent with our previous findings [2]. Disruption of HSPGs by shNDST1 also significantly reduced ERK activation (Figure 3B). These results suggest that both FAK and HSPGs play crucial roles in ERK activation regulating MMP-13 expression. The data also show that inhibition of FAK and removal of HSPGs might not be complete because flow can still induce ERK activation, but the levels of ERK activation were not significantly higher than no-flow control (time 0 without any other treatment case) except for the case of PF-228 treatment. These data are consistent with the data in Figures 1 and 2 showing complete attenuation of the MMP-13 expression response to flow after treatment with heparinase (Figure 1B), shNDST1 (Figure 1B), and shFAK (Figure 2A).

### HSPGs mediate interstitial flow-induced FAK and ERK activation

**Figure 4:**
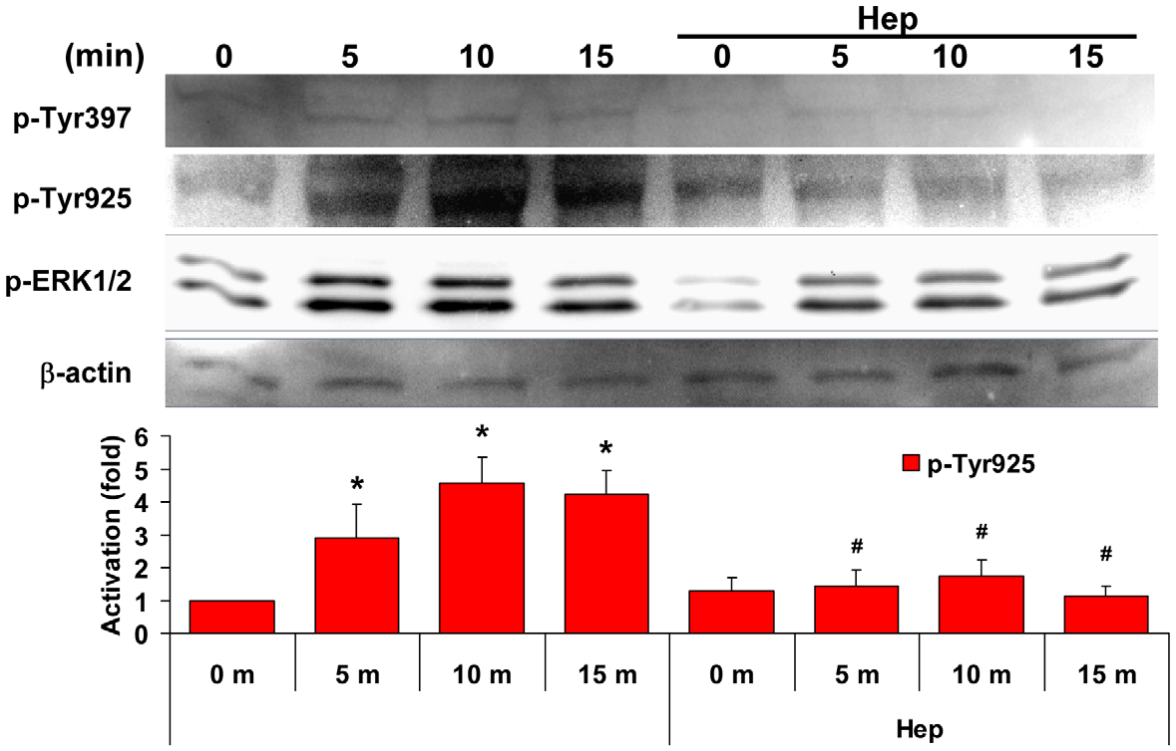
HSPGs mediate interstitial flow-induced activation of FAK Tyr925 and ERK. Cells in gels were pretreated with 6.7 IU/L of Hep for 2 h to remove cell surface HSPGs. Flow was induced for 0, 5, 10 or 15 min. Removal of HSPGs reduced FAK phosphorylation at Tyr397, but interstitial flow did not affect Tyr397 phosphorylation. Flow promoted Tyr925 and ERK phosphorylation, and cleavage of HSPGs blocked the flow effects. The gel panels are representative images from three independent experiments. Fold change values are the ratios of p-Tyr925 FAK over its internal control β-actin, and then normalized to no-flow control, shown in the bar graph. * P<0.05 vs time “0” in the non-Hep treated case; # P<0.05 vs corresponding time points in the non-Hep treated case. (Note: The signals of p-Tyr397 are rather weak and the background noises are rather strong due to low efficiency of protein extraction from SMCs in 3D collagen at the cell density we used. The levels of p-Tyr397 are thus not quantified.)

Knockdown of FAK or removal of HSPGs both abolished flow-induced ERK activation and MMP expression, suggesting that the mechanosensitive signaling pathways mediated by FAK and by HSPGs regulating ERK activation should be in a serial, not parallel pattern. Therefore, we hypothesized that HSPGs are flow sensors and signal transducers which sense and transmit flow stimuli to activate FAK and the downstream signaling cascade. To test this hypothesis, we eliminated cell surface HSPGs using heparinase and then investigated whether flow-induced activation of FAK and ERK was affected (Figure 4). Removal of HSPGs reduced FAK phosphorylation at Tyr397 and ERK activation in the no-flow case. Flow dramatically elevated phosphorylation of FAK Tyr925 and ERK; and these activations were markedly attenuated by cleavage of HSPGs. Flow appeared to play a lesser role in phosphorylation of FAK Tyr397. The results show that phosphorylation of FAK at Tyr397 correlates with baseline (no-flow) ERK activation (Figure 3) and baseline MMP expression (Figure 2), and seems to play a very minor role in flow-induced ERK activation and MMP expression; while activation of FAK at Tyr925 correlates with flow-induced ERK activation (Figures 3 and 4), MMP expression and cell motility (Figure 2). Disruption of HSPGs attenuated flow-induced activation of FAK and ERK (Figure 4) and knockdown of FAK blocked flow-induced activation of ERK (Figure 3), suggesting that HSPGs are mechanosensors mediating flow-induced FAK and downstream ERK signaling cascade activation and MMP expression.

### Integrin β1 but not HSPG is essential for cell spreading and integrin-mediated cytoskeletal organization is crucial for flow sensing

**Figure 5:**
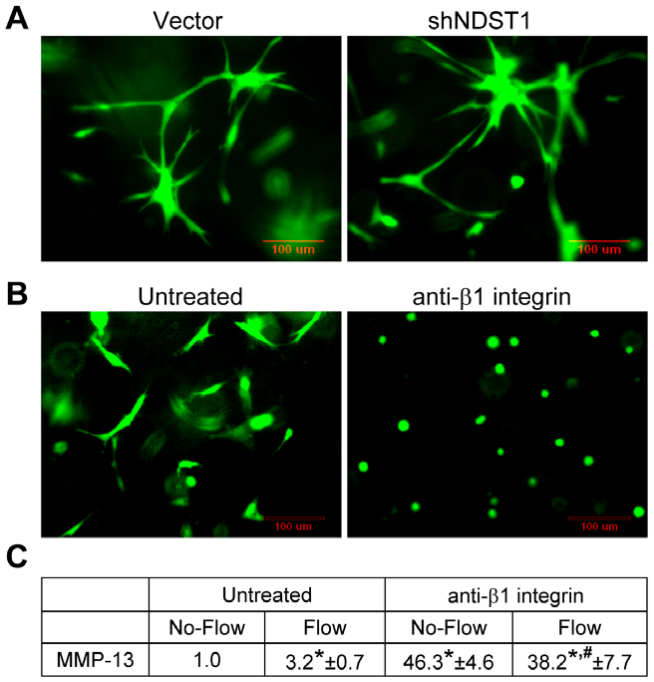
Integrin β1 but not HSPG is essential for cell spreading and integrin-mediated cytoskeletal organization is crucial for flow sensing. (**A**) Knockdown of NDST1 did not affect cell adhesion and spreading in collagen. (**B**) Integrin β1 antibody blocked cell spreading in collagen. (**C**) Blockade of β1 integrins altered baseline MMP-13 expression in the No-Flow case, and flow could not increase MMP-13 expression after blocking integrin β1; Data are mean ± SEM and are significantly different from the Untreated No-Flow case (*P<0.05, n = 3). There is no statistically significant difference between the Flow case and the No-Flow case when β1 integrins are blocked (# P>0.05, n = 3). To block β1 integrins, cells were pretreated with 40 µg/ml of integrin β1 antibody (NA/LE Hamster Anti-Rat CD29, clone Ha2/5, BD Pharmingen) for 15 min; cells were then suspended in collagen with 20 µg/ml Ha2/5 antibody and incubated for 12 h; the growth medium also contains 20 µg/ml Ha2/5 antibody. Cells were stained with Calcein AM in green. Images are the representative pictures from three independent experiments. For MMP-13 expression experiments, cells were incubated for 24 h in collagen gels followed by exposure to 6 h of flow.

After we disrupted HSPGs by knocking down of NDST1, we did not see significant changes in cell attachment and spreading either on a 2D surface (Figure 1A) or in 3D collagen after 24 h (Figure 5A), suggesting that integrin-based cell-matrix adhesions were still formed. Studies have shown that knockdown of syndecan-1 HSPGs inhibits cell attachment to collagen transiently and cells can still attach to collagen after 4 h [31], [39]. However, we also observed that cells could not spread out in collagen when β1 integrins were blocked (Figure 5B), suggesting that cell spreading through HSPG chain directly mediated attachments is negligible and integrins are indispensable for cell adhesion, spreading and maintaining cytoskeleton rigidity. Blockade of β1 integrins increased baseline MMP-13 expression in the No-Flow case, which is consistent with a previous report that blockade of α2β1 integrins induces MMP-1 expression in human fibroblasts [40]; however, flow could not induce MMP-13 expression when β1 integrins were blocked (Figure 5C). Therefore, we conclude that integrins provide a rigid cell cytoskeleton for mechanotransduction, while HSPGs sense interstitial flow to activate FAK and the downstream ERK cascade, eventually leading to an increase in MMP expression and cell motility.

## Discussion

Fluid flow in the tissue interstitium is very low due to the resistance of ECM fibrils and cells [41]. It has been shown, however, that such low flow can significantly affect cell physiology and function [1]–[3], [7], [13]–[15]. But, how cells sense this subtle flow in 3D remained largely unknown. Thus, the aim of this study was to determine the flow sensors on cells in 3D. We showed, for the first time, that, with contributions from integrins, HSPG-mediated activation of the FAK and downstream ERK signaling cascade plays the major mechanotransduction role in flow-induced rat MMP-13 expression and vascular SMC motility in 3D.

It has been shown that interstitial flow can promote tumor cell migration via autologous chemotaxis mechanisms [42]. In the present study, separation of the flow period from the migration period ensured that the effects of flow on the cell motility could not be interpreted as resulting from the convection of chemoattractant or other molecules produced by the suspended cells [1]. In addition, the flow velocity in this study is ∼0.5 µm/s; assuming the diffusion coefficient for cell secreted chemokines is ∼100 µm^2^/s and the cell radius is ∼5 µm, then according to Fleury et al. [42], the Peclet number is ∼0.025 which is rather small, suggesting that convective transport effects would be very small. Furthermore, in our preliminary study, we found that after exposure to 6 h of interstitial flow, followed by incubation with DMEM without PDGF-BB for 48 h, there were barely any migrated cells on the undersides of insert membranes observed, similar to that of no-flow controls without PDGF-BB as chemoattractant (Data not shown). This suggests that, due to our special experimental design, autologous chemotaxis mechanisms did not play a significant role in the study. Therefore, in this study we are able to distinguish the mechanical role of HSPG in flow sensing from its possible involvement in autologous chemotaxis mechanisms.

HSPGs are present over the entire cell surface, binding extracellular ligands and forming signaling complexes with receptors. The binding of cell surface HSPGs to ECM components can immobilize the PGs, enabling HSPG core proteins to interact with the actin cytoskeleton [22], [25]. Therefore, HSPGs can act as both coreceptors and mechanosensors in most ECM and cytoskeleton interactions. In the absence of integrins, binding of HSPGs to ECM via antibodies can support cell attachment and spreading through reorganization of the actin cytoskeleton and can mediate solid strain-induced mechanotransduction [22], [29], [36]. It has been suggested that HSPGs play important roles in EC and tumor cell migration or invasion [43], [44]. In the present 3D study, we showed that disruption of HSPGs by heparinase reduced MMP-13 expression and SMC motility in the no-flow control case and completely abolished flow-induced MMP-13 expression and cell motility (Figure 1). In contrast, a previous 2D study showed that HSPG disruption by heparinase enhanced EC migration by decreasing stress fibers and the size of focal adhesions, and increased migration under flow conditions [43], suggesting that HSPGs may play distinct roles in cell migration in 2D and 3D.

Appropriate cell-matrix adhesions are critical for cells and tissues to maintain function. Focal adhesions are macromolecular contact complexes between cells and ECM. FAK is a signaling molecule in the focal adhesion complex involved in integrin downstream signaling. Stimulation of integrins and many other cell surface receptors can cause FAK autophosphrylation at Tyr397, generating a binding site for Src family protein tyrosine kinases. Recruitment of Src family kinases induces FAK phosphorylation at Tyr925 which triggers Ras/MAPK cascade activation [45]. FAK plays a central role in mediating cell migration [38], [46], [47]. Shear stress can induce ERK activation dependent on FAK in 2D [48]. In this study, we showed that inhibition of FAK suppressed interstitial flow-induced MMP expression and cell motility (Figure 2) due to inhibition of ERK (Figure 3), suggesting that FAK is the cytoplasmic mediator of flow-induced ERK activation in 3D.

Shear stress can induce FAK phosphorylation at Tyr397 in 2D [48], [49], probably mediated by HSPGs on the cell's apical surface transmitting the shear force through the cytoskeleton to focal adhesions on the basal side [50], [51], where they are assembled for directional migration [43]. Cyclic strain can induce FAK phosphorylation at both Tyr397 and Tyr925 [52], [53], however, strain-induced ERK activation is mediated by FAK phosphorylation at Tyr925, not Tyr397 [53]. Interstitial flow significantly enhanced FAK phosphorylation at Tyr925 in 3D, which correlates with flow-induced MMP expression and ERK activation (Figures 2 and 4). Flow-induced ERK activation was mediated by FAK (Figure 3) and disruption of HSPGs abolished flow-induced FAK and ERK activation (Figures 3 and 4), but disruption of HSPGs did not affect cell spreading via integrins (Figure 5A), suggesting that flow sensing is mainly through HSPGs, not integrins. It has been shown that syndecan-1 can colocalize with α2β1 integrin and support integrin-mediated adhesion to collagen [31], [39] and syndecan-4 can cooperate with α5β1 integrin and mediate focal adhesion formation on fibronectin [54]. Syndecans then regulate organization of cortical actin and induce stress fiber formation at adhesion complexes [29], [31], [54], [55] and modulate FAK activation [35]. Since flow-induced mechanotransduction is mediated by FAK, the HSPGs that function as flow sensors in 3D might be directly located at the sites of cell-matrix adhesion or linked to matrix adhesion complexes via the cortical actin cytoskeleton, where these HSPGs are able to pass signals to FAK [32]. When cells are embedded in 3D, cell-matrix adhesions form all around the cell surface, however, the level of phosphorylated FAK is lower than on 2D [12], [18], suggesting that HSPG-mediated signaling may compensate for the reduced function from integrins.

To adhere to the ECM, integrins (α and β subunits range in size from 80 to 130 kDa) form αβ heterodimers and the extracellular domains directly attach to the binding sites in the ECM and cytoplasmic domains interact with the cytoskeleton [30]. The length of integrin-mediated adhesion is around 15 nm [56], [57]. When exposed to solid strains, integrin-based adhesions can be easily deformed due to the relative motion between the ECM and cell membrane, resulting in activation of integrin signaling. Therefore, integrin-mediated focal adhesions have been widely described to be mechanosensors for solid strain [58]. Unlike integrins, HSPGs (especially syndecans) contain a relatively short transmembrane core protein with several long and flexible HS-GAG chains extended into the extracellular space [30]. Monomeric syndecan core proteins range in size from 20 to 45 kDa [59]. In mediating cell adhesion, syndecans form stable homodimers and bind to the heparin/heparan sulfate binding sites in the ECM with the HS chains and the cytoplasmic domains on core proteins interact with the cytoskeleton [30]. Therefore, HSPG-mediated cell-matrix adhesion can be an alternative signaling pathway to the integrin signaling cascade [36]. Noting that collagen fibers are rather rigid and the pore size of collagen gels in vitro is around 0.5–1.0 µm [3], [60] and the space between two adjacent collagen fibers in the media of human aortas is greater than 50 nm (estimated from [61], [62]), there is plenty of space for HS chains (diameter <1 nm, [20], [33]) and even HSPG core proteins (diameter <10 nm, estimated from [63]) to move.

**Figure 6:**
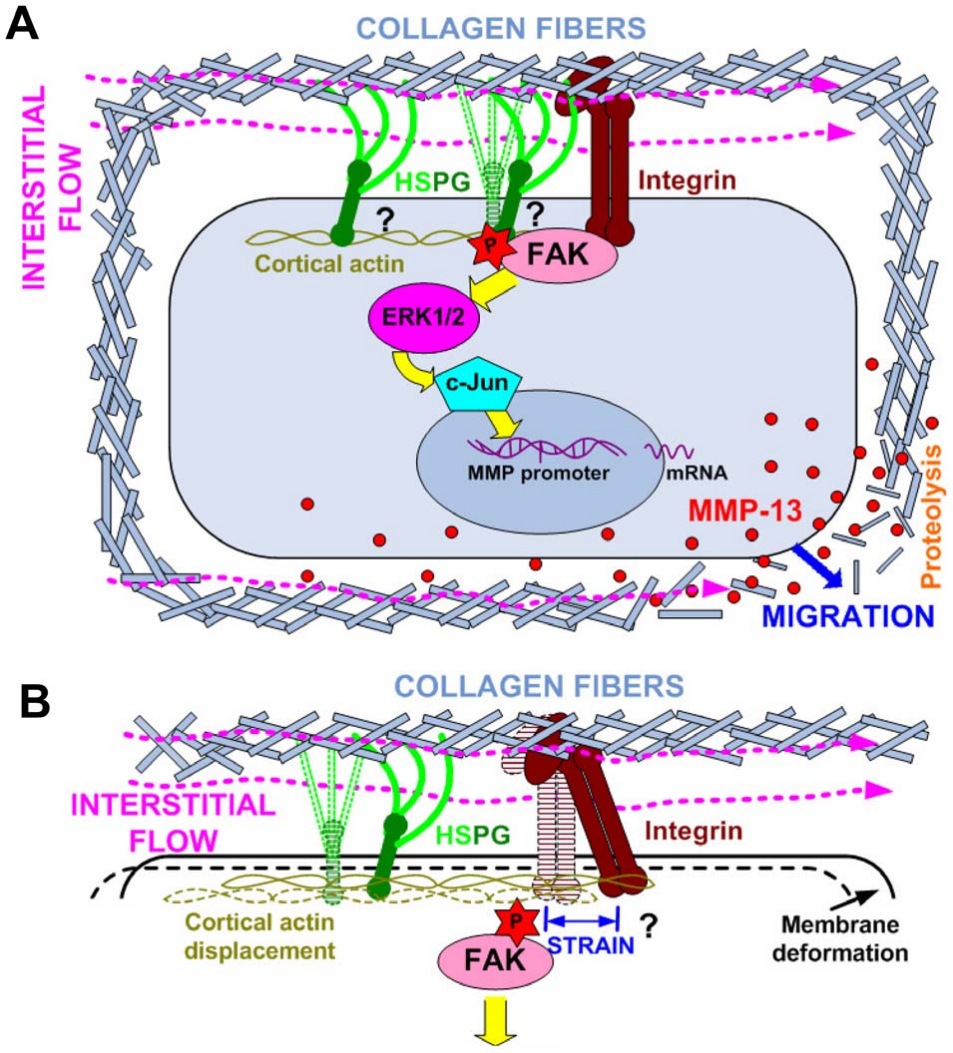
An interstitial flow mechanotransduction model and the roles of HSPGs and integrins in FAK activation in 3D. We propose that cell surface glycocalyx HSPGs are responsible for sensing interstitial flow. (**A**) HSPGs can interact with FAK either by colocalizing with integrin-mediated cell-matrix adhesion complexes, or by interacting with adhesion complexes through the cortical actin cytoskeleton, or both. Flow force causes HSPG deformation that is transmitted through the cell cytoskeleton to the adhesion complex, where the flow force triggers FAK-ERK-c-Jun-MMP-13 signaling axis. Elevated MMP expression promotes cell motility. HSPGs can be deformed as a result of flow forces on the flexible HS-GAG chains. Relatively rigid integrin-ECM bonds may not play a major role in direct flow sensing because there is not much strain between the cell membrane and the ECM; the presence of integrin-mediated cell-matrix adhesions is crucial for mechanotransduction. (**B**) Another possibility is that deformation of HSPGs induced by flow may also cause cortical actin displacement and cell plasma membrane deformation, which can actually generate a mechanical strain on integrins, leading to an activation of FAK-ERK axis. HSPGs may also transduce the flow signal to FAK activation through other chemical or mechanical connections (not shown in the model). Exactly how the flow force is transmitted through HSPG to activate FAK remains to be investigated. HSPG, integrin, cortical actin, and cell membrane shown in dashed lines represent the positions before flow and in solid lines indicate their positions while experiencing interstitial flow. (Note: MMP-13 protein release from cells should be nondirectional; To simplify the drawing, the release of MMP-13 is only shown on one side).

Based on our experimental results and the characteristics of integrins and HSPGs, we propose a model to summarize our mechanotransduction hypothesis, shown in Figure 6. The HS-GAG chains are rather long and flexible and may be easily deformed by shear flow, thus HSPG structures are more sensitive to interstitial flow than integrins. When exposed to interstitial flow, the flexible HS chains can be deformed causing HSPG core protein deformation that is transmitted to the cytoskeleton, leading to activation of the FAK signaling cascade. On the other hand, since integrin-mediated bonding is rather rigid, the flow-induced displacement (strain) of integrins may be much less than that of HSPGs, implying less mechanotransduction through integrins than HSPGs. However, cell spreading through HSPG alone is negligible and integrin-mediated adhesions are indispensable for maintaining cell cytoskeleton rigidity which is important for mechanosignal sensing and transduction [64]. We therefore speculate that, HSPGs play a major role in sensing interstitial flow and mediating mechanotransduction through FAK activation in 3D, by either colocalizing within integrin-mediated cell-matrix adhesion complexes (Figure 6A right) [65] or interacting with adhesion complexes through the cortical actin cytoskeleton (Figure 6A left) [55], [66], or both (Figure 6A). Another possibility is that deformation of HSPGs induced by flow may also cause cortical actin displacement and cell plasma membrane deformation, which can actually generate a mechanical strain on integrin-ECM bonds similar to a model suggested previously in osteocytes [67], and thus leading to an activation of FAK and ERK (Figure 6B). It may also be possible that HSPGs transduce the flow signal into FAK activation through other unknown connections (chemical or mechanical). Exactly how the flow force is transmitted through HSPG and then activates FAK remains to be further investigated.

Previously we have shown that interstitial flow-induced rat MMP-13 expression and vascular SMC, fibroblast and myofibroblast motility depend on activation of the ERK-c-Jun signaling pathway [1], [7] and surface glycocalyx HSPG-mediated ERK activation regulates fluid flow modulation of SMC and myofibroblast phenotypes [2]. In the present study, we further showed that in 3D, cell surface HSPG-mediated FAK phosphorylation is responsible for ERK activation although the specific HSPG core proteins responsible for force transmission to FAK remain to be determined. Synthesizing all of these observations, we propose, for the first time, that HSPG-mediated FAK activation is a mechanism for interstitial flow-induced mechanotransduction (Figure 6). Since interstitial flow can also induce tumor cell migration via an autologous chemotaxis mechanism [42], it would remain interesting to know whether HSPGs play any roles in this mechanism. We conclude that interstitial flow can, by a HSPG-mediated mechanism, in concert with integrin-mediated cell-matrix adhesions and cytoskeleton organization, induce activation of the FAK-ERK-c-Jun signaling axis, regulating MMP expression, vascular cell motility, and vascular cell phenotype in 3D. While this study was based on vascular cells and motivated by the response of an artery to injury, the mechanotransduction mechanism that we have proposed should be relevant to 3D flow effects on tissue morphogenesis, cancer cell metastasis and stem cell differentiation, and also have implications in tissue engineering.

## Materials and Methods

### Collagen gel preparation, flow experiment, and migration assay

As previously described [1], [7], rat vascular SMCs were suspended in 4 mg/ml collagen I (BD Science) (2.5×10^5^ cells/ml) and then loaded into culture inserts with 8 µm pores (BD Science). After 24 h of incubation, cells were subjected to interstitial flow driven by 1 cmH_2_O pressure drop (flow velocity: 0.5 µm/s; shear stress: ∼0.05 dyn/cm^2^) for various time periods according to the specific experimental designs. For FAK inhibition and HSPGs cleavage experiments, after 24 h of spreading, cells in gels were treated with either 10 µM PF-228 (Santa Cruz Biotechnology) or 6.7 IU/L heparinase III (IBEX Technologies, Montreal, Canada) in growth medium for 2 h and then exposed to flow. Flow medium contained either 10 µM PF-228 or 1 IU/L heparinase. (Note: The doses of PF-228 from 1 to 10 µM have been used in many other articles. We tested both 2.5 and 10 µM in our preliminary studies in 3D collagen. We observed that at both doses, flow-induced MMP-13 expression could not be completely abolished. Later we found out that flow-induced MMP-13 expression may be only partially dependent on FAK Tyr397. And also higher dose of PF-228 could affect cell growth (see Ref. [38]), therefore we decided to use 10 µM of PF-228 for this 3D study.)

To check the effect of flow on cell motility, as described previously [1], immediately after the flow period, 1 ml of 20 ng/ml PDGF-BB (Sigma) was added to each plate well to initiate migration to the bottom of the insert membrane. After 48 h of chemotactic incubation, the cells that had migrated to the undersides of the insert membranes were stained with Diff-Quik (Dade Behring), and five fields (100X) (1 center, 4 edges) were randomly picked for counting. The migration was then normalized to its No-Flow control case.

### Immunofluorescence staining

To stain HS-GAGs, cells in the plate wells were fixed with 4% paraformaldehyde for 15 min and blocked with 4% BSA in PBS for 30 min, and followed by incubating with primary antibody HepSS-1 (US Biological) (1∶200 dilution in PBS with 4% BSA) for 2 hours and then secondary antibody Alexa Fluor 350 goat anti-mouse IgM (invitrogen) (1∶100 dilution) for 2 hours at room temperature. Finally cells were mounted by mounting medium containing propidium iodide (PI) (Vector Laboratories). To visualize cell morphology in 3D collagen gels, cells were stained with Calcein AM (invitrogen) (1∶200 in growth media).

### Protein extraction and Western blotting

Protein samples were collected and western blotting was performed as described previously [7]. Collagen gels were washed once with ice-cold PBS, then 2X lysis buffer was added immediately to the gels followed by sonication for 30 seconds on ice. The 2X lysis buffer was composed of 2X RIPA buffer (300 mM NaCl, 2% NP-40, 100 mM Tris, 0.2% Brij 35, 2 mM EDTA, pH 7.5) with a supplement of 2X protease inhibitor cocktail (Roche Diagnostics), 2X phosphatase inhibitor cocktail (Roche Diagnostics), 2 mM activated Na_3_VO_4_, and 2 mM PMSF. Lysates were centrifuged in a microfuge (12,000 g for 1 hour at 4°C), and then the supernatants were collected and the remaining gel pellets were discarded. The supernatants were concentrated using Centrifugal Filter Units (Millipore). Protein concentrations in supernatants were evaluated using Protein Determination Kit (Cayman Chemical). The protein samples were then boiled for 5 minutes after mixing with 4X sample buffer (400 mM Tris-HCl, 8% SDS, 40% glycerol, 0.04% bromphenol blue, and 20% β-mercaptoethanol, pH 6.8) and stored at −80°C. Protein samples were loaded onto 10% Tris-HCl Ready Gels (Bio-Rad). After electrophoresis, proteins were transferred to PVDF membranes (Bio-Rad) and blocked at room temperature with 2% Enhanced Chemiluminescence (ECL) Advance Blocking Agent (Amersham, GE Healthcare) in TBS-T. The membranes were incubated overnight with a 1∶1000 dilution of a specific rabbit primary antibody (monoclonal antibodies: ERK1/2, phospho-ERK1/2; polyclonal antibodies: FAK, phospho-FAK (Tyr397), phospho-FAK (Tyr925), and β-actin. All antibodies were purchased from Cell Signaling), followed by a 1.5-h room temperature incubation with an ECL horseradish peroxidase (HRP)-linked anti-rabbit IgG antibody (1∶1000) (Amersham, GE Healthcare). The proteins on PVDF membranes (Bio-Rad) were then detected using Immobilon Western Chemiluminescent HRP Substrate (Millipore) and the ChemiDoc XRS system with the Quantity One software (Bio-Rad). Some membranes were stripped using Restore™ Plus Western Blot Stripping Buffer (Thermo Scientific Pierce) for subsequent detections.

### RNA interference

Two FAK shRNAs and one control shRNA (gift from Dr. Tadashi Yamamoto) were used for FAK silencing [68]. The following sequences were used:

FAK#1, 5′-GGTCCAGACCAATCACTAT-3′;

FAK#2, 5′-GCAGTTTGCCAACCTTAAT-3′;

and a control sequence, 5′-TTCTCCGAACGTGTCACGT-3′;

and the vector was pSIREN-RetroQ [68]. FAK#1 and #2 shRNAs were mixed together in equal amount and cotransfected into the cells. To disrupt heparan sulfate biosynthesis, a rat NDST1 shRNA was used (Origene, MD). The target sequence for rat NDST1 was:

5′-CTTACTGTGCTCCTCAATCCTATCAGCGT-3′,

which was subcloned into pGFP-V-RS vector. For transfection, 15 µg of each plasmid (shFAK, p-SIREN-C, shNDST1, and pGFP-V-RS) per T-75 flask were used. The transfections were conducted using Lipofectamine™ LTX and PLUS™ reagents (Invitrogen) as previously described [7]. The cells were used for various experiments 2 days after transfection.

### RNA extraction and gene expression analysis

Cells in collagen were directly lysed by Trizol reagent and the insoluble materials were removed by centrifugation at 12,000×g for 10 minutes at 4°C. Chloroform was added for phase separation followed by RNA purification using the RNeasy Mini Kit (Qiagen). After reverse transcription (RT), polymerase chain reaction (RT-PCR) was performed using the following protocol: pre-denaturation at 95°C for 5 minutes, then either 30 cycles (for MMP-13) or 28 cycles (for GAPDH) of denaturation at 94°C for 35 seconds, annealing at 55°C for 35 seconds, and extension at 72°C for 35 seconds, followed by a final extension at 72°C for 10 min. The amplified products were separated by electrophoresis in 2.5% agarose gels and photographed under UV light in the presence of ethidium bromide (EB). Quantitative real time PCR (RT-qPCR) was also performed for MMP-13 expression on the ABI PRISM® 7000 sequence detection system (Applied Biosystems) using the following protocol: 15 minutes at 95°C followed by 45 cycles of 30 seconds at 95°C, 30 seconds at 55°C, and 30 seconds at 72°C. Rat interstitial collagenase MMP-13 (GenBank Locus: NM_133530) primer sequences were:

forward, 5′-TCTGACCTGGGATTTCCAAAAG-3′ (1124–1145);

reverse, 5′-GTCTTCCCCGTGTCCTCAAA-3′ (1194–1175).

Rat NDST1 (GenBank Locus: NM_024361.1) primer sequences were:

forward, 5′-GATGACCCGGTGGCCCTAAA-3′ (2607–2626);

reverse, 5′-TCTGTTCGCAGCAGTTTGCC-3′ (2797–2778).

Primer sequences of GAPDH were listed previously [1], [7].

### Data Analysis

Results are presented as mean ± SEM. Data sets were analyzed for statistical significance using a Student's t-test with a two-tailed distribution, and P<0.05 was considered statistically significant.

## Supporting Information

Figure S1 **Disruption of smooth muscle cell surface glycocalyx heparan sulfate by heparinase III and NDST1 knockdown.**
(PDF)
