# Supplementary material for: Heparan Sulfate Proteoglycans Mediate Interstitial Flow Mechanotransduction Regulating MMP-13 Expression and Cell Motility via FAK-ERK in 3D Collagen
Source: PLoS One. 2011 Jan 5;6(1):e15956. doi: 10.1371/journal.pone.0015956 (PMC3016412; doi:10.1371/journal.pone.0015956)
Supplement: Figure S1 — Disruption of smooth muscle cell surface glycocalyx heparan sulfate by heparinase III and NDST1 knockdown. (PDF) [file pone.0015956.s001.pdf]

Shi ZD et al., Supporting information: Figure S1

Disruption of cell surface glycocalyx heparan sulfate by heparinase III and NDST1 knockdown

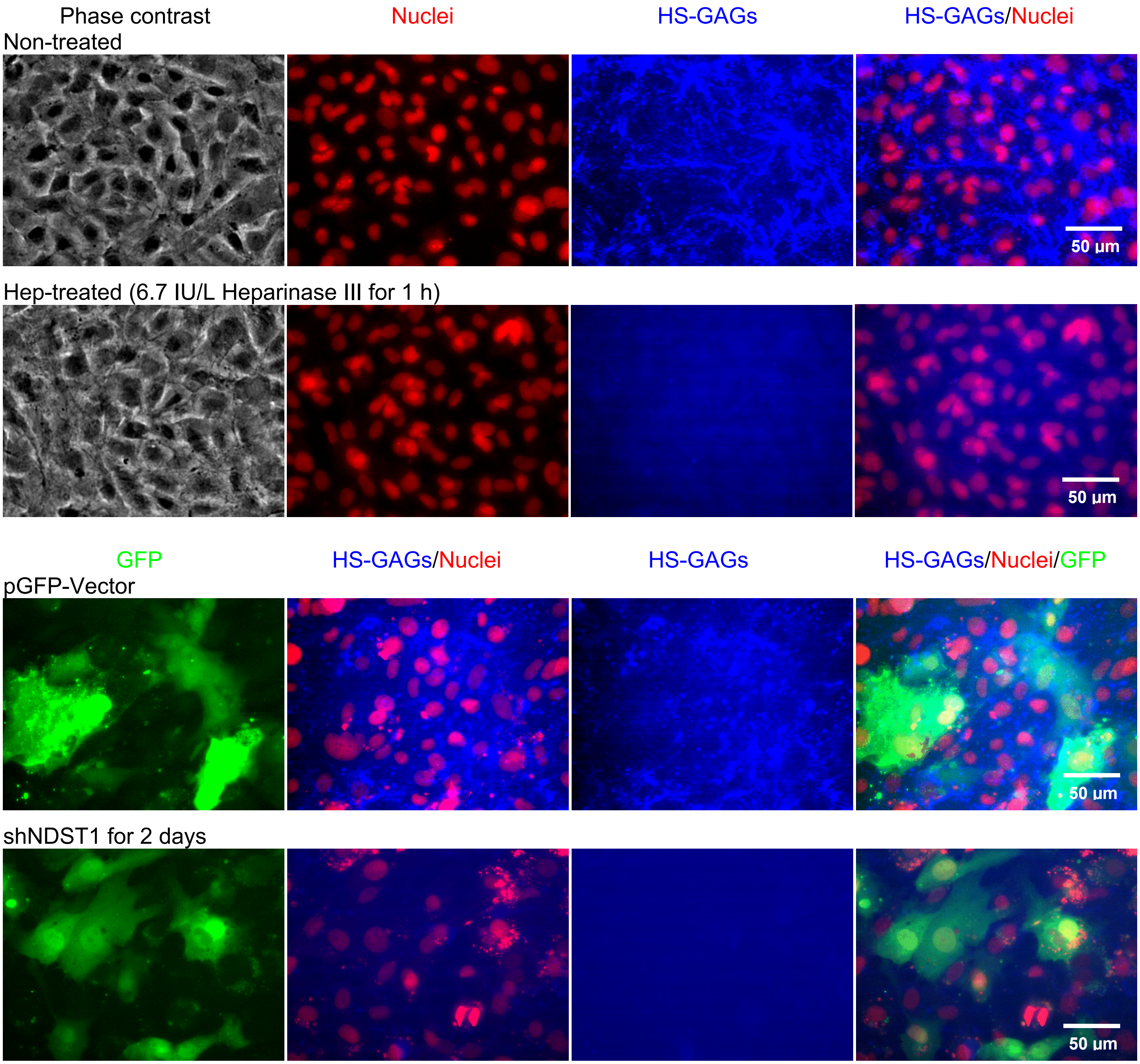

Green: GFP; Blue: Alexa Fluor 350; Red: PI

Besides immunostaining for evaluation of HS-GAG production after NDST1 knockdown, we also assessed the efficacy of NDST1 knockdown by RT-qPCR and we observed 40~50% inhibition of NDST1 mRNA after 1 to 3 days with 15 μg plasmid per T-75 flask. This knockdown efficiency was enough to significantly reduce heparan sulfate production and attenuate flow-induced cell motility and MMP expression. The cells were used for experiments 2 days after transfection.
